# The social context of gender-based violence, alcohol use and HIV risk among women involved in high-risk sexual behaviour and their intimate partners in Kampala, Uganda

**DOI:** 10.1080/13691058.2015.1124456

**Published:** 2016-01-19

**Authors:** Jasmine Schulkind, Martin Mbonye, Charlotte Watts, Janet Seeley

**Affiliations:** ^a^ Department of Global Health and Development, London School of Hygiene and Tropical Medicine, London, UK; ^b^ Social Science Programme, Medical Research Council/Uganda Virus Research Institute Uganda Research Unit on AIDS, Entebbe, Uganda

**Keywords:** Sex workers, HIV, alcohol, gender-based violence, Uganda

## Abstract

This paper explores the interaction between gender-based violence and alcohol use and their links to vulnerability to HIV-infection in a population of women and their regular male partners in Kampala, Uganda. Data derive from 20 life history interviews (10 women and 10 men). Participants were drawn from a cohort of women at high risk of sexually transmitted infection (including HIV). Six of the women were current or former sex workers. Findings reveal that life histories are characterised by recurrent patterns of gender inequity related to violence, limited livelihood options and socioeconomic disadvantage. Overall, findings suggest women are able to negotiate safer sex and protect themselves better against abuse and violence from clients than from their intimate partners, although the status of men as ‘client’ or ‘partner’ is transitory and fluid. Among male respondents, alcohol led to intimate partner violence and high levels of sexual-risk taking, such as engagement with sex workers and reduced condom use. However, male partners are a heterogeneous group, with distinct and contrasting attitudes towards alcohol, condom use and violence. Actions to address gender-based violence need to be multi-pronged in order to respond to different needs and circumstances, of both women and men.

## Résumé

Cet article explore l’interaction entre la violence basée sur le genre et la consommation d’alcool, et leurs liens avec la vulnérabilité au VIH des femmes, dans un groupe composé de femmes vivant à Kampala et de leurs partenaires masculins réguliers. Les données sont extraites de 20 entretiens axés sur l’histoire de vie (10 femmes et 10 hommes). Les participantes ont été recrutées dans une cohorte de femmes fortement exposées aux infections sexuellement transmissibles (y compris le VIH). Parmi ces femmes, six avaient été ou étaient des travailleuses du sexe. Les résultats révèlent que les histoires de vie sont caractérisées par des modèles récurrents d’inégalité entre les sexes liée à la violence, à des choix de vie limités et à des problèmes socio-économiques. D’une manière générale, les résultats suggèrent que les femmes ont la capacité de négocier le sexe à moindre risque et de mieux se protéger des abus et de la violence exercés par les clients que de ceux exercés par les partenaires intimes, bien que le statut de « client » et celui de « partenaire » soient transitoires et fluides. Concernant les répondants masculins, la consommation d’alcool conduisait à la violence entre partenaires intimes et à des niveaux élevés de prise de risque sexuel, reflétés par la fréquentation des travailleuses du sexe et une diminution de l’usage du préservatif. Cependant, les partenaires masculins sont un groupe hétérogène dans lequel les attitudes vis-à-vis de l’alcool, de l’usage du préservatif et de la violence sont différentes, voire contrastées. Les actions visant à réduire la violence fondée sur le genre doivent être basées sur plusieurs approches pour mieux répondre aux différents besoins et circonstances, aussi bien des femmes que des hommes.

## Resumen

El presente artículo examina la relación existente entre la violencia basada en el género y el consumo de bebidas alcohólicas, así como sus vínculos con la vulnerabilidad a la infección por vih, en una población de mujeres y sus parejas masculinas habituales en Kampala. La información al respecto deriva de 20 entrevistas centradas en investigar las historias personales de diez mujeres y diez hombres. Las participantes fueron seleccionadas de una cohorte de mujeres con alto riesgo de contraer una infección transmitida sexualmente (incluyendo el vih). Seis de ellas son sexoservidoras actualmente o lo fueron en el pasado. Los hallazgos revelan que sus historias personales muestran patrones recurrentes caracterizados por inequidad de género relacionada con la violencia, escasas opciones en cuanto a medios de vida y padecimiento de desventajas socioeconómicas. En general, estos hallazgos sugieren que las mujeres demuestran mayor habilidad con sus clientes que con sus parejas íntimas cuando se trata de negociar el sexo más seguro y de protegerse contra el abuso y la violencia, aun cuando la clasificación de los hombres como “clientes” o “parejas” es transitoria y fluida. Por otra parte, entre los participantes masculinos el alcohol constituye un factor que deriva en violencia contra la pareja íntima, así como en altos niveles de riesgo sexual, entre los que se incluye tener relaciones con sexoservidores y disminuir el uso de condones. Sin embargo, las parejas masculinas constituyen un grupo heterogéneo que manifiesta actitudes distintas y contrastantes en torno al consumo de alcohol, al uso de condones y a la violencia. Por ello, las acciones encaminadas a afrontar la violencia con base en el género deben ser multifacéticas, a fin de que puedan responder a distintas necesidades y circunstancias, tanto de las mujeres como de los hombres.

## Introduction

Despite continuing efforts to address the epidemic, HIV remains a leading cause of mortality in sub-Saharan Africa. One of the key drivers of HIV in sub-Saharan Africa is sex work, with far higher rates of HIV infection among female sex workers compared to the general population. In Kampala, Uganda, for example, HIV prevalence among female sex workers is estimated to be 37% (Vandepitte et al. 2011). However, despite widespread recognition of the importance of female sex workers as a key population in sub-Saharan Africa, HIV/STI prevention interventions targeting women in sex work have been relatively small-scale and with limited impact (Scorgie et al. 2012). Research within this field has predominantly focused on the epidemiological importance of female sex workers in HIV transmission with less focus on the social context of women’s lives. Crucially the role of regular partners of female sex workers in determining HIV risk has been largely neglected (Huynh et al. 2014; Panchanadeswaran et al. 2008).

Despite global efforts to address gender-based violence, such as the United Nations Secretary General’s UNiTE to End Violence against Women campaign, gender-based violence remains one of the greatest threats to women’s health and wellbeing worldwide (World Health Organisation 2013). Studies from Uganda, South Africa and India document how female sex workers are particularly vulnerable to high levels of violence from both intimate partners and clients (Mbonye et al. 2012; 2014; Panchanadeswaran et al. 2008; Pauw and Brener 2003; Schwitters et al. 2015), reducing their ability to negotiate safer sex. However, there remains limited research on men’s perspectives on gender-based violence and gender inequities – both as male clients of sex workers and as their intimate partners (Huynh et al. 2014; Scorgie et al. 2012).

A small number of qualitative studies from India (Karnataka and Chennai) and Uganda focus on intimate partner violence among female sex workers (Huynh et al. 2014; Mbonye et al. 2012; Panchanadeswaran et al. 2008). These document the complexities in defining their intimate relationships; ‘clients’ are men who pay money in exchange for sex, although it is common for women to form an emotional attachment to a regular client. This client may then become a regular partner who provides economic support and a potential exit out of sex work (Mbonye et al. 2012). Importantly, studies demonstrate that female sex workers are often less able to negotiate condom use with intimate partners (Mbonye et al. 2012, 2013; Rutakumwa et al. 2015) and may be at greater risk of violence from partners than clients (Huynh et al. 2014; Panchanadeswaran et al. 2008).

Although various models exist to unpick the relationship between alcohol use, gender-based violence and HIV infection (Fritz et al. 2002; Jewkes et al. 2010), no explicit overarching framework exists to fully explain this complex interaction. Furthermore, existing models do not look specifically at the individual risks alcohol and violence pose for female sex workers. Research from Kampala has found high levels of excessive alcohol use among women and their clients, associated with risky sexual behaviour, including inconsistent condom use (Mbonye et al. 2013; Vandepitte et al. 2011). In this paper, we provide a deeper understanding of the social context of gender-based violence among women at high-risk of HIV-infection and their intimate male partners in Kampala, with a view to informing future interventions. This paper is based on data drawn from a qualitative research project focusing on the structural drivers of HIV infection.

## Research methods

### Cohort recruitment

This study was nested within a clinic-setting providing support to women at high-risk of HIV infection and their male partners. The project was established by the Medical Research Council/Uganda Virus Research Institute, Uganda, Research Unit on AIDS in 2008. Women attending the clinic who are involved in high-risk sexual behaviour are eligible for enrolment into the main research cohort (Vandepitte et al. 2011). Women identified as ‘high-risk’ are those engaging in sex work or jobs such as bar work, restaurant work and karaoke entertainment, and women with multiple concurrent partners. As of July 2014, there were over 2,600 female participants and about 140 male partners who were attending the clinic and participating in different research projects. The clinic is located on the outskirts of Kampala, in a densely populated area of low cost housing. Women receive free general and reproductive healthcare, including STI testing and treatment, medical care for their children as well as health-education counselling on HIV risk and alcohol intake. The women are encouraged to bring their male partners to the clinic, where they are also invited to take part in research activities.

Sex work is a broad concept with boundaries that are difficult to define (Harcourt and Donovan 2005). In this paper we have used the term only for women who self-identify as sex workers. However, many of the women do engage in transactional sex for money or gifts, but do not perceive themselves to be sex workers. Among those identifying as sex workers there are important differences with regards to levels of alcohol use and risk of violence, as described elsewhere (Mbonye et al. 2013). Notably, street-sex workers in Kampala appear to be more exposed to stigmatisation and violence from clients and the public in comparison to bar-based sex workers.

### Data collection

As a part of a qualitative study of the structural drivers of HIV infection, in-depth life history interviews were collected from participants by experienced social science interviewers. Participants for interview were recruited by a statistician. They were systematically selected from the clinic population to include a proportion of those uninfected by HIV, those with an initial infection and those superinfected with HIV. Interviewers were blind to the HIV status of the interviewee prior to the interview (although many interviewees chose to discuss their status during the interview). By September 2014, a total of 58 women and men had participated in this project. This paper focuses on the most recent data from 10 women and 10 men, collected between July 2013 and September 2014. Interviewees were selected from the cohort as individuals without knowledge of the relationships between men and women attending the clinic. We did not conduct formal interviews with clinic staff. However, background information on the running of the clinic was gleaned from daily interactions with counsellors and nurses at the clinic.

Each participant was interviewed on three separate occasions in Luganda, the local language, enabling rapport to be developed between the participant and the interviewer. Due to the sensitive nature of the topics discussed, interviewers were matched to the same gender as the participant. Interviews were conducted at the clinic or at another setting, if preferred by the participant. Participants were given 5,000 shillings (roughly $2) as a transport refund and a soft drink during the interviews. The interviews were semi-structured and lasted around 60 to 90 minutes each. The initial interview was largely unstructured, leaving participants to tell their life history. This was then built upon in subsequent interviews, which were used to explore more sensitive issues including relationship history, experiences of sex work and attitudes towards HIV infection and condom use, once rapport had developed. Tape recorders were not used at the request of respondents. Instead, the interviewers, skilled in qualitative data collection, took notes during the interview, which they then wrote up into detailed transcripts immediately following each interview. This allowed them to recall and include quotations from the participants, some of which are used in this paper. Each interview was then translated into English by the interviewers themselves, who were bilingual.

Ethical approval for this project was provided by the Scientific and Ethics Committee of the Uganda Virus Research Institute and the Uganda National Council for Science and Technology. The ethics committee of the London School of Hygiene and Tropical Medicine provided additional clearance for the analysis upon which this paper is based. Informed consent was obtained and confidentiality was stressed throughout the interviews. Participants were offered counselling at the clinic to address psychological effects of, for example, discussions around domestic violence. This follows the WHO ethical and safety recommendations for domestic violence research (World Health Organisation 2001). Data were anonymised and pseudonyms are used in this paper.

### Methodological approach

An ecological approach was taken to understand the predictors of gender-based violence in this cohort, based on Heise’s (1998) integrated ecological framework. This model incorporates the personal, microsystem, exosystem and macro-level factors that explain why some women are at greater risk of violence and abuse or why certain men perpetrate gender-based violence. Factors at the personal level include experience of violence in childhood or individual male attitudes towards gender-based violence. Microsystem refers to influences from within the relationship, family or close peer network, whilst the exosystem represents the wider social environment. Macrosystem factors refer to broad societal beliefs. They include gender inequities, male dominance and social condonement of violence against women. A woman’s risk of gender-based violence can be viewed as a complex interplay between these different levels.

### Thematic data analysis

A largely inductive approach was used to code the data based on a method developed by Auerbach and Silverstein (2003). The transcripts from women and men were coded separately into themes and sub-themes manually. The analysis was an iterative process, with the themes continually reworked as the transcripts (55 female, 63 male) were reread and discussed with the interview team. Relevant text from each interview was highlighted and arranged into groups of repeating ideas expressed by more than one of the participants.

## Results

### Sample demographics

All the participants were Ugandan, living in the area near the clinic and most had migrated from areas outside of Kampala. The majority were of low socioeconomic status and educational status. Most occupied cheap one-roomed, concrete, houses close to small bars and lodges. The average age of the female and male participants interviewed for this study was 31 years and 37 years, respectively. All of the men and women had at least one child and the majority were in multiple concurrent relationships, reflecting the ‘high-risk’ nature of this population. Although polygamy is relatively common in Uganda (Seeley 2012), few participants reported being in a formal polygamous marriage. However, a number of participants reported being involved in extramarital relationships, as previously described in this population (Mbonye et al. 2012; Rutakumwa et al. 2015). Of the 10 women, 8 voluntarily disclosed their HIV status: 5 were HIV-positive and 3 said they were HIV-negative. Of the male partners, 5 reported that they were HIV-positive and 5 HIV-negative. Of the 10 women, 5 were engaged in sex work at the time of the study, while 1 had left sex work. Of these, 2 were bar-based sex workers, 2 street-based and the other 2 worked as karaoke singers but regularly engaged in sex work as part of their employment. All of these women engaged in other forms of employment alongside sex work, such as street food vending or housework. The other four women did not self-identify as sex workers but all reported having multiple sexual partners who provided them with gifts and financial support.

## Early life history

### Female participants

Strong themes of poverty, lack of education and gender inequities are depicted in the women’s early life histories. The majority describe losing one or both parents in childhood, often to HIV-infection. None of the women completed education past primary level, which most gave as a reason for early sexual relations and confinement to low-skilled employment in later life. Physical and sexual violence was also pervasive in women’s early lives, with 5 of the participants describing physical violence and abuse in the school or home environment: ‘we were always beaten for being late at school’ (Jennifer, 31 years, market stall seller). One woman described facing daily threats of violence while living on the street:

Staying on streets was so hard. There were boys who were so violent to us. They would beat us up and sometimes made attempts to rape us. (Susan, 27 years, street-based sex worker)

This interplay of early life challenges and reduced employment options appears to facilitate participants’ entry into early sexual relationships and sex work.

### Male participants

Many of the themes from the women’s early lives were mirrored in interviews with their intimate partners: poverty, early loss of parents in childhood, violence in school and ‘mistreatment’ at home by stepparents and family members. Male participants had a higher level of education compared to female participants, with eight of the men educated past primary level and one participant who had attended university. Over half of the men had begun casual employment while still in school, for example as motor vehicle mechanics, doing building work and food vending, which they continued into adult life.

A striking common narrative seen in five of the men’s accounts is the early influence of high-risk sexual behaviour. Three of the men describe being introduced to the world of nightclubs and sex work by older men as young teenagers. Matthew, 26 years, explained how ‘I used to bring him [his boss] sex workers and this was normal to me’, and he himself engaged with sex workers and alcohol from a young age. Multiple partnerships also emerged as a continual theme throughout the interviews, with eight of the men describing their father as having children from multiple wives. It is through this backdrop that we begin to understand the men’s attitudes to alcohol use, violence and high-risk sexual behaviour.

## Gender-based violence sex work

### Structure and social organisation of sex work in Kampala 

While some participants viewed sex work as their main employment, others engaged in transactional sex informally, alongside other forms of employment:

I just remained with a few sexual partners who would meet some of my daily needs like rent and food. (Mary, 35 years, bar-based sex worker)

An implicit hierarchy exists within sex work, as described previously in this population (Mbonye et al. 2013). Street sex workers possessing a lower social status and perceived higher risk behaviour compared to older, more experienced bar-based workers, as expressed by one bar-based sex worker:

Those who line up on the streets or purely into sex work are the ones who go to any place they find even if it means having sex in the corridor. (Mary, 35 years, bar-based sex worker)

Karaoke and bar work were both intimately linked to the sex work industry: three of the participants described being coerced into sex work by bar or karaoke managers.

### Structure of sex work in relation to gender-based violence

Women faced the constant threat of violence, which, according to clinic staff, women perceive as an inevitable part of their lives. However, participants were able to protect themselves to some extent by, for example, avoiding clients’ homes, as this placed them at a greater risk of violence and abuse:

I refused to go with some when I saw that they were hooligans because with those ones, you can reach their homes and they instead beat you up and take everything else that you have. (Susan, 27 years, street-based sex worker)

Many street-based sex workers and managers of sex-work lodges in Kampala run an informal security system, with some lodge owners employing security men, known as *Kanyama men* (or body builders), to stand outside the lodge. Many lodge owners also paid policemen to intervene if any clients became violent.^1^

### Violence in day-to-day lives

Violence pervades the lives of the women interviewed. Within the bars where many women worked as barmaids, fights between men (who were also clients) were common: ‘they would fight whenever they got drunk and they would break the chairs’ (Hannah, 21 years, karaoke-based sex worker). These men were in turn violent towards sex workers: ‘these men can take and beat you up after robbing you’ (Susan, 27 years, street-based worker). Immediate triggers for client-perpetrated violence centre on use of alcohol, disputes over pay and possession over a particular sex worker:

When I slept with him, he thought I would always sleep with him so, on that day, he found me taking booze with another man and he waited for me outside and beat me up. (Margaret, 38 years, former bar-based sex worker)

Women also describe being abused and sexually assaulted as barmaids and hotel workers. The interviews suggest women perceive abuse as inevitable in their work, as depicted by one barmaid discussing sexual harassment:

Since they are our customers, I don’t care so much because I am used to it and after all, [there is] nothing they take from me. (Hannah, 21 years, karaoke-based sex worker)

Some women were also faced with abuse from managers and the police:

The group leader started connecting men to us and a man would go through her [the group leader] so, even if you didn’t want, you had to accept by force …. In sex work, the problem was always the police. I was arrested for a number of times and would be detained for around two days. (Susan, 27 years, street-based sex worker)

In general, however, the transcripts contain very few accounts of interactions with the police, though discussion with the counsellors suggested that sexual harassment and violence from the police was commonplace and taken as inevitable by the women, with police demanding bribes or sex from street-based sex workers. Fights between street sex workers were also frequent, leading on occasion to police involvement. These were commonly attributed to high levels of substance use leading to aggressive behaviour and fierce territorial tensions:

The chemicals [inhaled drugs] women used always resulted into fights …. Women always ended up with big cuts on their bodies and some were even imprisoned. (Susan, 27 years, street-based sex worker)

Some women portrayed themselves as exercising high levels of control over interactions with clients: ‘These days when I get a man, if he does not want to use a condom, I tell him to go away’ (Chloe, 35 years, karaoke-based sex worker). However, elsewhere in the same interview the woman reported finding herself in situations in which she had little or no control: ‘I struggled to escape him, but he overpowered me and did what he pleased’ (Chloe, 35 years, karaoke-based sex worker), suggesting that the control was aspirational rather than the norm.

## Relationships

### Context of intimate relationships

#### Female participants

Relationships with men among the participants varied, with distinct roles played by different partners. Economic support was central to the formation and context of relationships, with many men preventing their partners from engaging in employment*.* Notably, the distinction between client and partner was not clear-cut in a setting where regular clients frequently become partners. As a result, it was not always easy to distinguish between client and partner violence.

Women described a particular type of man in the interviews. Certain partners were far more likely to be controlling, violent, engage in high-risk sexual behaviour and refuse condom use. Mary, when describing condom use with her first partner says, ‘I was the one who always told him to use them and he would always carry them with him … I was very assertive and never feared to say what I wanted.’ In a subsequent relationship, however, her partner refused condoms: ‘This one had truly loved me and when I talked about condoms, he changed the tone and asked me whether I was a prostitute.’ This is the same partner who ‘turned into a beating and quarrelsome person’ and who she suspected of infecting her: ‘I have been through a lot, the man mistreated me and, on top of that, he infected me with HIV!’ (Mary, 35 years, bar-based sex worker).

#### Male participants

For men, many relationships were casual and formed under the influence of alcohol: ‘He met her during the times when they used to take a lot of alcohol as they went out but he also had many other partners’ (Matthew, 26 years, businessman), while others began more formally as marriage, and the paying of a bride price. For other men, the boundary between client and partner was less clear: ‘I consider her as my wife because I don’t pay her when we have sex’ (Eric, 28 years, market stall owner).

A number of the men described how their first sexual encounter had been with an older women:

This lady used to take great care of me while at home and used to entice me with a lot of gifts … the woman caressed me and I ended up having sex with her. On my first time, I ejaculated prematurely and she then taught me how to hold back. (John, 46 years, builder)

In this case, it was clearly the older woman taking control of sexual relations, acting as a teacher to the young man. The relationship could be interpreted as the man engaging in transactional sex.

Economic dependence within relationships emerged as a key theme, with the majority of male participants reporting that they provided material benefits for their partner. Men described their ideal partner as being ‘well-behaved’ and ‘respectful’. These perceptions of an ideal wife act as macro-level drivers of gender-based violence, reducing women’s power within the relationship and condoning the use of violence to control women.

Several important differences emerged between relationships with sex workers and those with women for whom transactional sex was not their main means of earning money. Men perceived women who engaged in sex work as possessing a greater degree of economic independence in comparison with most female partners, and thus the power dynamic within the relationship shifted. Eric (28 years, market stall owner) described being financially dependent on his partner: ‘the most important thing is that she is so close and easy for me and she can cover in for me financially any time when I am in trouble’. However, economic independence did not equate to greater equality within the relationship. Eric went on to say: ‘she is a sex worker and I hear people call her harlot but she seduced me.’ Clinic staff noted that sex workers were often not respected by their partners due to high levels of social stigma, placing them at higher risk of violence.

All of the men interviewed had multiple concurrent partners, with many reporting high levels of partner change: ‘I used to have sex with a new person nearly every week’ (Eric). Multiple partners were perceived to be a status symbol for men, with interviewees reporting taking a second partner to ‘express my masculinity’ or ‘to show off’. This contrasts with men’s attitudes to their partners’ promiscuity, which several stated that they highly disapproved of and described as a trigger for violence. This demonstrates deep-rooted gender inequities at the macro-system level.

### Condom use in relationships

#### Female participants

Diverse experiences of condom use emerged from the transcripts, reflecting distinct patterns of power within relationships. Some women reported consistent condom use, while others expressed a complete inability to negotiate safe sex: ‘I do not know how I can avoid HIV in marriage, it’s only God to help me’ (Jennifer, 31 years, market stall seller). Women clearly understood their risk of HIV but, as a result of either gender inequities within relationships or a desire to distinguish between intimate partners and clients, did not discuss issues around condom use with regular partners.

#### Male participants

Reported condom use among men was also highly variable and was not necessarily related to the HIV status of participants, as has been described previously among this population (Rutakumwa et al. 2015). Some participants insisted they consistently used condoms, while others perceived condoms to be for casual relationships, not marriage: ‘since I had been living with her for some time, I got used to her and ended up having unprotected sex’ (Patrick, 27 years, market stall seller). Men’s attitudes towards condom use and high-risk sexual behaviour reflected social constructs of masculinity at the macro-level: ‘I was so brave and had nothing to fear’ (Eric, 28 years, market stall owner). As clients of sex workers, however, the majority of men reported that sex workers insisted on condom use.

## Intimate partner violence and abuse

### Female participants

Abuse by a male intimate partner was a common narrative in all the interviews. Participants recounted numerous episodes of physical violence and persistent threats of violence from past and current partners: ‘the man started behaving like a mad man’ (Mary, 35 years, bar-based sex worker); ‘he started quarrelling and promised to set us ablaze’ (Margaret, 38 years, former bar-based sex worker).

In co-habiting relationships in particular, respondents described how physical violence commonly occurred alongside emotional and economic abuse. Mary, whose husband was physically violent towards her during pregnancy, described how she also suffered neglect at that time: ‘my husband was silent and rendered no support’. Six of the participants recounted recurrent episodes of neglect or emotional abuse, mostly when they were co-habiting with a partner. Participants also reported a greater likelihood of abuse if their partner had taken alcohol, indicating a link between heavy alcohol consumption and perpetration of violence: ‘when my partner drinks alcohol, he starts shouting at the top of his voice that I brought HIV in the family’ (Margaret, 38 years, former bar-based sex worker). A number of women reported how persistent abuse within relationships had significant effects on their mental and physical health: ‘I would fail to sleep and even lost so much weight due to stress’ (Jennifer, 31 years, market stall seller).

Sexual violence or coercion within marriage was not often mentioned in the interviews. Counsellors, however, recount frequent reports of physical violence, marital rape and emotional abuse by women attending the clinic. The counsellors and interviewers described how intimate partner violence was frequently under-reported because it is so normalised within Uganda and because women fear that if they report violence they will face further abuse from their partners.

### Male participants

At the level of the exosystem, men described an environment of violence and alcohol use in their day-to-day lives, with fights between men as commonplace. However, individual participants had contrasting attitudes towards intimate partner violence. Many men admitted to perpetrating physical violence as a form of controlling or punishing their partner if she was ‘promiscuous’ or did not ‘do her domestic chores’. Matthew, aged 26, described fights over money when he had taken alcohol: ‘she may think you have drunk all the money yet it may be your friends that have bought for you alcohol.’ At this, he would end up beating her when she ‘bothers him a lot’. However, John suggested that, despite cultural norms condoning violence and a highly violent environment, perpetration of intimate partner violence is not inevitable:

If you can’t contain the anger caused by the woman, walk away and return later on when the anger is gone. (John, aged 46 years, builder)

## Alcohol and substance use

### Female participants

Many of the lodges in which sex-work was conducted are attached to bars where women sit and wait for clients to buy them drinks. This can, however, put women at particular risk of abuse if the women and/or their clients become drunk:

Some men would refuse to pay us after sex and they would ask us for the number of bottles we took and then the amount of money they cost. And you couldn’t argue with them because you would be drunk already. (Mary, 35 years, bar-based sex worker)

Although street-based sex workers may be offered drinks by clients, most drank and used drugs while on the streets as a coping mechanism ‘to be able to withstand street life’ (Susan, 27 years, street-based sex worker). In particular, alcohol was seen to protect women against the cold, give them courage to deal with clients and cope with social stigma. Most respondents reported drinking heavily in addition to smoking pipes with tobacco or marijuana: ‘I used to take *waragi* [local gin], beers and would even lose count of the number of bottles I had taken’ (Jennifer, 31 years, market stall seller).

### Male participants

Alcohol and other drugs are an equally important part of the male partners’ lives. Men report taking mostly *waragi* and *njaga* (marijuana) and the majority drank socially in bars and clubs where there was a strong peer influence:

The most common place we used to hang out at was [anonymised] pub and my friends used to buy me a lot of alcohol added to involving freely with women. (Matthew, 26 years, businessman)

### Alcohol, violence and high-risk sexual behaviour

#### Female participants

A complex relationship between alcohol and safe sex negotiation emerged from the transcripts. Some women perceive alcohol as necessary to negotiate condom use: ‘Alcohol would give me courage to rudely ask men to put on light and put on condoms while [I was] watching’ (Mary, 35 years, bar-based sex worker). However, clear patterns emerge in several transcripts between alcohol, risky sexual behaviour and vulnerability to abuse:

I got so excited whenever I drank … I went with every kind of man … I drank too much and could not even stand on my own feet. Men had sex with me without my knowledge. (Susan, 27 years, street-based sex worker)

#### Male participants

Heavy drinking and high-risk sexual behaviour pervade the majority, although not all of the interviews with the men. In particular, men described having high numbers of casual relationships and low condom use when drunk: ‘I used to like alcohol a lot and yet when you get drunk you don’t screen any one.’ (Matthew, 26 years, businessman).

A relationship between alcohol, unsafe sex and violence was also evident:

When I get drunk and she pushes me away, I go and get a sex worker and satisfy my desires …. Other risks with alcohol are fighting and getting injured and also pushing me into sexual relationships because when you get drunk, you get all the courage to confront any woman (Matthew, 26 years, businessman)

Alcohol was also a powerful risk factor for perpetration of intimate partner violence:

When he takes *waragi*, he gets a bad temper and always fights with the wife when he takes it. He said he even some times beats her and doesn’t remember that the following day. (Interviewer notes for Robert, 28 years, commercial motorcycle driver)

## Discussion

The drivers of gender-based violence in this cohort reflect a complex interplay of factors at different levels of the ecological framework (Heise 1998). For women engaging in sex work, the findings of this study are consistent with previous research identifying the importance of the local sex work environment, or exosystem, in shaping risk in relation to alcohol use, violence and HIV/STI infection (Mbonye et al. 2014; Schwitters et al. 2015). In particular, the transcripts reveal how bar-based sex workers were at risk of excessive drinking and abuse due to normative practices of clients ‘owning’ a woman once he has bought her alcohol. This finding links to research from South Africa that describes how alcohol is frequently used as a currency for transactional sex (Watt et al. 2012). Street-based sex workers in sub-Saharan Africa are generally perceived to be of lower social status and at greater risk of violence and abuse (Scorgie et al. 2012). However, informal security arrangements within the lodges offered some form of protection among this population of sex workers. In contrast, respondents who worked as barmaids or karaoke singers, in addition to engaging in sex work, appeared to be highly controlled by managers and thus less able to exert individual agency and insist on condom use when selecting clients. This underscores the need for a detailed understanding of the local organisation of sex work when designing effective interventions. Personal factors, in particular a woman’s individual assertiveness or decision-making, appear to play an equally important role in determining condom use and risk of violence from clients. Importantly, high levels of economic need among sex workers, referred to as ‘survival sex’ in the literature, reduce individual participants’ autonomy to refuse potentially violent clients (Harcourt and Donovan 2005).

In terms of women’s risk of violence and abuse from their partners, widespread patriarchal beliefs pervaded the transcripts of the men interviewed, reflecting deep-rooted gender inequities at a macrosystem level. What emerge are individual narratives of male dominance, perpetration of intimate partner violence and high-risk behaviour in particular transcripts, embodying social constructs of masculinity. Although certain high-risk behaviours, such as concurrent partnerships, were common among the male partners interviewed, these men cannot be treated as a homogenous group with distinct and contrasting attitudes towards alcohol, condom use and gender-based violence. This demonstrates how perpetration of gender-based violence by male participants is strongly influenced by diverse factors at the personal and microsystem level.

Alcohol use was deeply embedded in the lives of the women and men attending the clinic. Among male respondents, alcohol led to increased disinhibition, perpetration of intimate partner violence and high levels of sexual-risk taking, such as high numbers of casual partners, engagement with a sex worker and reduced condom use. This reflects the central role of alcohol in the exosystem. The data supports previous findings in Uganda and South Africa that individual perpetration of intimate partner violence is associated with controlling behaviours, multiple concurrent partnerships and alcohol use among men (Abramsky et al. 2011; Jewkes et al. 2010; Schwitters et al. 2015). The findings highlight the difficulty in disentangling the relationship between alcohol use, violence and risky sexual behaviour in this population, and the need for an overarching framework.

The microsystem is central to determining women’s risk of abuse within relationships. The data suggests that women are better able to negotiate safe sex and protect themselves against abuse from clients than from intimate partners, echoing other findings from Uganda and India (Gysels, Pool, and Nnalusiba 2002; Panchanadeswaran et al. 2008). Women’s risk of violence appears to change throughout relationships, supporting findings from a recent WHO multi-country study on women’s health and domestic violence that co-habiting women worldwide are at greater risk of intimate partner violence compared with those not living with their partners (Abramsky et al. 2011). There is, however, a large amount of fluidity between clients and regular partners. Therefore, intimate partner violence and client violence cannot be treated as separate entities when designing interventions.

Despite the pervasiveness of sexual violence and coercion in Uganda (Ahikire and Mwiine 2013), and among sex workers in particular (Schwitters et al. 2015), it was reported far less than physical and emotional abuse in the interviews. Sexual violence by intimate partners in particular was rarely mentioned. This likely reflects the social stigma surrounding domestic sexual violence. The interviews show high levels of overlap between emotional, economic and physical abuse from partners, corroborating previous research stressing the need for integrated interventions that target the constellation of abuse faced by women (Abramsky et al. 2011; Krug et al. 2002). The high prevalence of non-physical abuse and neglect among participants reinforces a growing recognition of the importance of emotional abuse within relationships, which has received less attention due to challenges in measuring and defining non-physical abuse (Abramsky et al. 2011).

Thus far, sex worker-targeted HIV prevention strategies have largely neglected women’s risk of HIV from their intimate partners, notably pervasive intimate partner violence and low levels of condom use with regular partners (Panchanadeswaran et al. 2008). The findings presented in this paper suggest that community-wide interventions, such as SASA!, a community mobilisation intervention in Kampala challenging gender power relations and gender-based violence (Abramsky et al. 2014), which shift deep-rooted societal norms around intimate partner violence and male dominance in relationships, would benefit female sex workers. There is a clear need to involve intimate partners in gender-based violence prevention programmes. Among male participants there is an evident need to address the link between excessive alcohol use, intimate partner violence and risky sexual behaviour. Considering the role of alcohol in the study setting, a bar-based intervention, led by peers within existing social networks, may be effective (Fritz 2009).

Echoing research from similar populations in sub-Saharan Africa (Agha and Chulu Nchima 2004; Gysels, Pool, and Nnalusiba 2002; Schwitters et al. 2015), the findings from this study highlight the need to address the illegality of sex work. The criminalisation of sex work at the macro-level stigmatises women within their communities, placing them at a high risk of violence and abuse from police, partners and clients and vulnerability to HIV. Despite high prevalence of intimate partner violence and client violence, none of the women in this cohort reported these incidents to the police.

The interviews provide critical insights into the perspectives of partners of women, which have received little attention. However, this study has some important limitations. The interviews were not linked so it was not possible to compare the responses of individual women and their partners. Our study sample is based on clinic attendees who were willing to participate in the study. Additionally male participants were recruited from those who came voluntarily to the clinic after being invited by their partners. Thus, interviews may not be representative of the attitudes and experiences of other women at high risk of HIV-infection and their male partners more generally. As mentioned earlier, formal interviews with clinic staff were not carried out. Participants referred to interviewers as *musawo*, meaning health worker. This is a term often used by participants referring to staff involved in health research. The team make it clear to participants that they are not clinic staff or medically trained but the setting and the association with the clinic could lead to some social desirability bias in the answers given – misreporting of condom use in interviews, for example, is a common challenge (Scorgie et al. 2012). While the study took place within a specific location and with a small group of participants, and interpretation of findings needs to be considered within that context, the findings also suggest contextual factors common across sub-Saharan Africa. For example, key themes that emerge from the research include socioeconomic disadvantage, alcohol abuse and exposure to gender-based violence.

## Conclusion

The past decade has seen a growing recognition of the need to address persistent gender inequities and gender-based violence if we are to reduce women’s vulnerability to HIV infection. Among female sex workers and other high-risk women, gender inequality, gender-based violence and alcohol use continue to place women at an unacceptably high risk of violence and HIV/STI infection from clients and partners. This study shows how, within the social context of female sex work in Kampala, these issues are intricately interwoven and cannot be treated in isolation. In seeking to contribute towards a greater understanding of the relationship between gender-based violence, alcohol and HIV risk among a key population in the epidemic, it highlights the difficulties in defining ‘sex worker’, ‘client’ and ‘partner’ in this social context and the implications for HIV and gender-based violence prevention. These findings, and those of previous research, point towards the critical need to involve partners and clients in interventions in the context of pervasive gender inequities. A multi-pronged approach, integrating alcohol reduction, gender transformative interventions, violence prevention and HIV services with a strong structural focus, is urgently needed.

## Funding

The research was funded under the UK Medical Research Council (MRC) and the UK Department for International Development (DFID) under the MRC/DFID Concordat agreement.

## Note

1. The local sex work environment is described more fully elsewhere (Mbonye et al. 2013).
